# A phase I study of the safety, tolerability, and pharmacokinetics of contezolid acefosamil after intravenous and oral administration in healthy Chinese subjects

**DOI:** 10.1128/aac.00796-23

**Published:** 2023-10-30

**Authors:** Haijing Yang, Yi Jin, Hailin Wang, Hong Yuan, Jingjing Wang, Size Li, Yingying Hu, Huahui Yang, Xin Li, Hong Liang, Jufang Wu, Guoying Cao, Jing Zhang

**Affiliations:** 1 Phase I Clinical Research Center, Huashan Hospital, Fudan University, Shanghai, China; 2 Key Laboratory of Clinical Pharmacology of Antibiotics, Ministry of Health, Shanghai, China; 3 National Clinical Research Center for Geriatrics, Shanghai, China; 4 Shanghai MicuRx Pharmaceutical Co., Ltd., Shanghai, China; 5 Institute of Antibiotics, Huashan Hospital, Fudan University, Shanghai, China; Providence Portland Medical Center, Portland, Oregon, USA

**Keywords:** contezolid acefosamil, MRX-4, contezolid, oxazolidinone, healthy subject, safety, pharmacokinetics

## Abstract

Contezolid acefosamil (also known as MRX-4), a prodrug of contezolid, is under development for treatment of multidrug-resistant Gram-positive bacterial infections. A phase I single ascending dose (SAD) and multiple-dose placebo-controlled study was conducted to assess the safety, tolerability, and pharmacokinetics (PK) of contezolid acefosamil in healthy Chinese subjects following intravenous (IV) and oral administration. Adverse events (AEs) and PK parameters were assessed appropriately. All subjects (*n* = 70) completed the trial. Overall, 67 cases of treatment-emergent adverse events (TEAEs) were observed in 49.1% (27 of 55) of the subjects receiving contezolid acefosamil. All TEAEs were mild in severity. No serious AEs or deaths were reported. After IV SAD (500–2,000 mg), the corresponding *C*_max_ of the active drug contezolid increased from 1.95 ± 0.57 to 15.61 ± 4.88 mg/L, AUC_0–inf_ from 40.25 ± 10.12 to 129.41 ± 38.30 h·mg/L, median *T*_max_ from 2.00 to 2.75 h, and mean *t*_1/2_ from 13.33 to 16.74 h. Plasma contezolid reached steady state on day 6 after multiple IV doses, with an accumulation ratio of 2.20–2.96. Oral SAD of 500 and 1,500 mg resulted in contezolid *C*_max_ of 8.66 ± 2.60 and 37.10 ± 8.66 mg/L, AUC_0–inf_ of 30.44 ± 7.33 and 162.36 ± 47.08 h·mg/L, and median *T*_max_ of 2.50 and 2.98 h. Contezolid reached steady state on day 5 after multiple oral doses of 1,500 mg without significant accumulation. Contezolid *C*_max_ and AUC_0–inf_ increased with the dose of contezolid acefosamil. The good safety and PK profiles in this SAD and multiple-dose study can support further clinical development of contezolid acefosamil.

## INTRODUCTION

The emergence of multidrug-resistant Gram-positive bacteria, including methicillin-resistant *Staphylococcus aureus*, vancomycin-resistant *Enterococcus*, and penicillin-resistant *Streptococcus pneumoniae*, poses great challenges to clinical treatment and also a threat to public health (1 – 3). The prevalence of drug-resistant Gram-positive bacteria is high in China. Data from the China Antimicrobial Surveillance Network showed that in 2022, the prevalence of MRSA and methicillin-resistant *Staphylococcus epidermidis* was 28.7% and 82.2%, respectively (www.chinets.com). Currently, the antibiotics commonly used for treating drug-resistant Gram-positive bacterial infections are usually associated with safety concerns (4). Vancomycin is associated with serious side effects such as nephrotoxicity, hypotension, and allergic reactions (5). Daptomycin may lead to myopathy and rhabdomyolysis (6). Tigecycline is associated with coagulopathy (7, 8), pancreatitis, and hepatotoxicity (9). Linezolid, the first oxazolidinone antibiotic approved by the US Food and Drug Administration (FDA), may cause thrombocytopenia and myelosuppression. Tedizolid, as a second-generation oxazolidinone antibiotic, did not show a significant advantage in myelosuppression in a real-world comparative safety study (10). These safety issues have to some extent limited the clinical use of these important antimicrobial agents (11, 12), which make the development of new drug-resistant antibacterial agents particularly urgent.

Contezolid (also known as MRX-I) is a fully synthetic oxazolidinone antimicrobial agent, independently developed by Shanghai MicuRx Pharmaceutical Inc (MicuRx). It was approved for treating multidrug-resistant Gram-positive bacterial infections in China in 2021 (13). Contezolid should be taken with meals or within 30 minutes after meals because food can enhance contezolid absorption. Contezolid is mainly excreted via urine and feces after being metabolized into various metabolites, most of which are excreted within 24 h after administration. The cumulative excretion of contezolid in urine and feces is less than 5% of the oral dose (13). Contezolid has shown some advantages over linezolid in terms of comparable efficacy (13, 14) and significantly lowers incidence of myelosuppression (13, 15). However, the poor solubility of contezolid has impeded the development of injectable dosage forms. Oral formulations of contezolid alone cannot meet the needs of patients with severe clinical infections, especially for those who cannot tolerate or take oral medications. Therefore, contezolid acefosamil (also known as MRX-4), a prodrug of contezolid, was developed (16). Contezolid acefosamil is available in both tablets for oral administration and injectable formulation (lyophilized powder for injection) due to its good solubility. Contezolid acefosamil is also developed by MicuRx in China. Contezolid acefosamil *per se* has no antibacterial activity. It is rapidly metabolized into the active drug contezolid *in vivo* via the intermediate product MRX-1352. Contezolid is mainly degraded into MRX-1320 and excreted in urine. Neither MRX-1352 nor MRX-1320 shows antimicrobial activity. Contezolid acefosamil was bioequivalent to contezolid in preliminary rat/mouse infection models (16, 17). In mouse models of systemic and thigh infections caused by multiple Gram-positive pathogens, contezolid acefosamil administrated either orally or intravenously demonstrated high antibacterial efficacy similar to linezolid. Moreover, the antibacterial efficacy of oral contezolid acefosamil was comparable to that of intravenous administration (18). A phase I single/multiple ascending dose study in healthy subjects receiving intravenous (IV) and oral contezolid acefosamil and a phase II study in patients with skin and skin structure infections were completed in the US (unpublished data).

In order to bridge with the data in the US and facilitate the approval of contezolid acefosamil in China and other countries simultaneously, a phase I clinical trial was conducted in healthy Chinese subjects. This placebo-controlled phase I study aimed to evaluate the safety, tolerability, and PK characteristics of contezolid and related metabolites after intravenous and oral administration of single ascending dose (SAD) and multiple doses of contezolid acefosamil in healthy Chinese subjects.

## RESULTS

### Demographics

**Fig 1:**
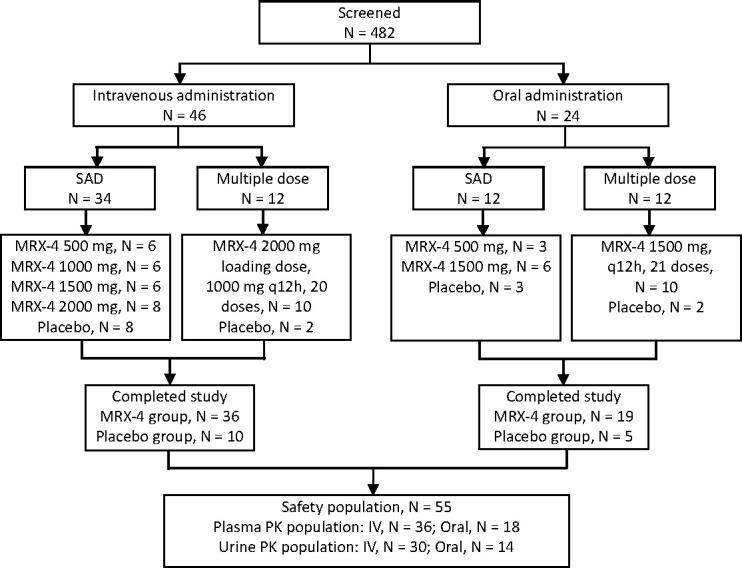
Disposition of study participants. IV, intravenous; MRX-4, contezolid acefosamil; PK, pharmacokinetic; SAD, single ascending dose.

A total of 482 subjects were screened, and 70 subjects were enrolled and randomized. All of the 70 subjects completed the study. A total of 55 subjects received contezolid acefosamil and were included in safety analysis (IV administration, *n* = 36; oral administration, *n* = 19). Plasma PK population included 54 subjects (IV administration, *n* = 36; oral administration, *n* = 18; one subject was excluded due to vomiting). Urine PK population included 44 subjects (IV administration, *n* = 30; oral administration, *n* = 14; data were not available for the subjects receiving 500-mg dose. The subjects were also excluded due to nausea and vomiting) (Fig. 1). The gender, age, and body mass index (BMI) of subjects were generally balanced among various dose cohorts (Table 1).

**TABLE 1:** Demographics and baseline characteristics of the study subjects^*a*^

		Intravenous administration of contezolid acefosamil or placebo								
		Single dose						Multiple dose		
Variable	Statistic	500 mg (*n* = 6)	1,000 mg (*n* = 6)	1,500 mg (*n* = 6)	2,000 mg (*n* = 8)	Placebo (*n* = 8)	Total (*N* = 34)	2,000/1,000 mg (*n* = 10)	Placebo (*n* = 2)	Total (*N* = 12)
Gender										
Male/female	*n*	3/3	4/2	3/3	4/4	3/5	17/17	5/5	1/1	6/6
Age (years)	Mean (SD)	30.3 (5.01)	29.0 (7.80)	28.5 (4.32)	31.4 (3.25)	29.0 (3.12)	29.7 (4.62)	30.6 (5.58)	32.5 (3.54)	30.9 (5.21)
Race										
Asian	*n* (%)	6 (100.0)	6 (100.0)	6 (100.0)	8 (100.0)	8 (100.0)	34 (100.0)	10 (100.0)	2 (100.0)	12 (100.0)
Non-Asian	*n* (%)	0 (0.0)	0 (0.0)	0 (0.0)	0 (0.0)	0 (0.0)	0 (0.0)	0 (0.0)	0 (0.0)	0 (0.0)
Height (cm)	Mean (SD)	161.78 (8.72)	166.92 (8.38)	163.75 (11.28)	162.23 (7.55)	164.20 (12.75)	163.71 (9.55)	160.95 (6.82)	160.30 (5.80)	160.84 (6.42)
Weight (kg)	Mean (SD)	61.26 (7.27)	65.11 (6.01)	59.78 (9.58)	63.44 (6.72)	62.03 (10.73)	62.37 (8.03)	58.11 (7.91)	59.25 (0.57)	58.30 (7.17)
BMI (kg/m^2^)	Mean (SD)	23.35 (1.32)	23.40 (1.82)	22.17 (1.37)	24.09 (1.79)	22.85 (1.24)	23.20 (1.58)	22.39 (2.22)	23.10 (1.45)	22.51 (2.07)

		Oral administration of contezolid acefosamil or placebo						
		Single dose				Multiple dose		
Variable	Statistic	500 mg (*n* = 3)	1,500 mg (*n* = 6)	Placebo (*n* = 3)	Total (*N* = 12)	1,500 mg (*n* = 10)	Placebo (*n* = 2)	Total (*N* = 12)
Gender								
Male/female	*n*	2/1	2/4	2/1	6/6	6/4	0/2	6/6
Age (years)	Mean (SD)	21.7 (5.51)	27.7 (6.68)	28.0 (5.20)	26.3 (6.20)	27.9 (3.57)	31.5 (6.36)	28.5 (4.01)
Race								
Asian	*n* (%)	3 (100.0)	6 (100.0)	3 (100.0)	12 (100.0)	10 (100.0)	2 (100.0)	12 (100.0)
Non-Asian	*n* (%)	0 (0.0)	0 (0.0)	0 (0.0)	0 (0.0)	0 (0.0)	0 (0.0)	0 (0.0)
Height (cm)	Mean (SD)	173.93 (2.48)	165.60 (6.26)	164.33 (11.72)	167.37 (7.74)	166.54 (6.74)	153.70 (1.56)	164.40 (7.90)
Weight (kg)	Mean (SD)	70.68 (5.32)	61.66 (11.05)	63.65 (7.62)	64.41 (9.28)	64.48 (6.40)	52.08 (7.67)	62.41 (7.88)
BMI (kg/m^2^)	Mean (SD)	23.35 (1.27)	22.37 (2.77)	23.58 (1.84)	22.92 (2.18)	23.24 (1.65)	22.02 (2.81)	23.03 (1.78)

^*a*^ BMI, body mass index; *n*, number of subjects SD, standard deviation.

### Safety evaluation

Overall, 67 cases of treatment-emergent adverse events (TEAEs) were observed in 49.1% (27 of 55) of the subjects receiving contezolid acefosamil, specifically in 14 (38.9%) of the 36 subjects receiving IV administration of contezolid acefosamil and 13 (68.4%) of the 19 subjects taking contezolid acefosamil orally.

A total of 29 cases of drug-related adverse events (AEs) were reported, specifically, in 4 (11.1%) of 36 subjects after receiving contezolid acefosamil intravenously and 9 (47.4%) of 19 subjects receiving oral contezolid acefosamil (1 subject in the single-dose cohorts and 8 subjects in the multiple-dose cohort) (Table 2). The AEs were mainly decreased white blood cell count and decreased neutrophil count in the subjects receiving IV contezolid acefosamil, and gastrointestinal disorders (nausea and vomiting) in the subjects receiving oral contezolid acefosamil. The drug-related AEs reported in >10% of subjects were mainly gastrointestinal symptoms, including nausea (26.3%), vomiting (10.5%), and abdominal distension (10.5%).

**TABLE 2:** Summary of drug-related TEAEs after intravenous and oral administration of contezolid acefosamil in healthy Chinese subjects^*a*^

	Intravenous administration							Oral administration				
	Single dose				Multiple dose	Placebo (*n* = 10)	Total (*N* = 46)	Single dose		Multiple dose	Placebo (*n* = 5)	Total (*N* = 24)
Type of TEAE	500 mg (*n* = 6)	1,000 mg (*n* = 6)	1,500 mg (*n* = 6)	2,000 mg (*n* = 8)	2,000/1,000 mg (*n* = 10)			500 mg (*n* = 3)	1,500 mg (*n* = 6)	1,500 mg (*n* = 10)		
Drug-related TEAE	0	0	2 (33.3)	1 (12.5)	1 (10.0)	0	4	0	1 (16.7)	8 (80.0)	0	9
Gastrointestinal disorder												
Nausea	0	0	0	0	0	0	0	0	0	5 (50.0)	0	5
Vomiting	0	0	0	1 (12.5)	0	0	1	0	1 (33.3)	2 (20.0)	0	3
Abdominal distension	0	0	0	0	0	0	0	0	0	2 (10.0)	0	2
Abdominal pain	0	0	0	0	0	0	0	0	0	1 (10.0)	0	1
Nervous system disorder												
Taste disorder	0	0	0	0	0	0	0	0	0	3 (30.0)	0	3
Dizziness	0	0	0	0	0	0	0	0	0	1 (10.0)	0	1
Investigations												
White blood cell deceased	0	0	2 (33.3)	0	1 (10.0)	0	3	0	0	0	0	0
Neutrophil count decreased	0	0	1 (16.7)	0	1 (10.0)	0	2	0	0	0	0	0
Serum bilirubin increased	0	0	0	0	0	0	0	0	0	1 (10.0)	0	1
Serum creatinine increased	0	0	0	0	0	0	0	0	0	1 (10.0)	0	1

^*a*^ Data are presented as number (%) unless otherwise specified. *n*, number of subjects; TEAE, treatment-emergent adverse event.

All of the TEAEs in this study were mild in severity and resolved spontaneously without treatment. No deaths, other serious adverse events (SAEs), significant AEs, or other AEs leading to early withdrawal from this trial were reported. No concomitant medication or treatment was used.

### Plasma PK parameters

**Fig 2:**
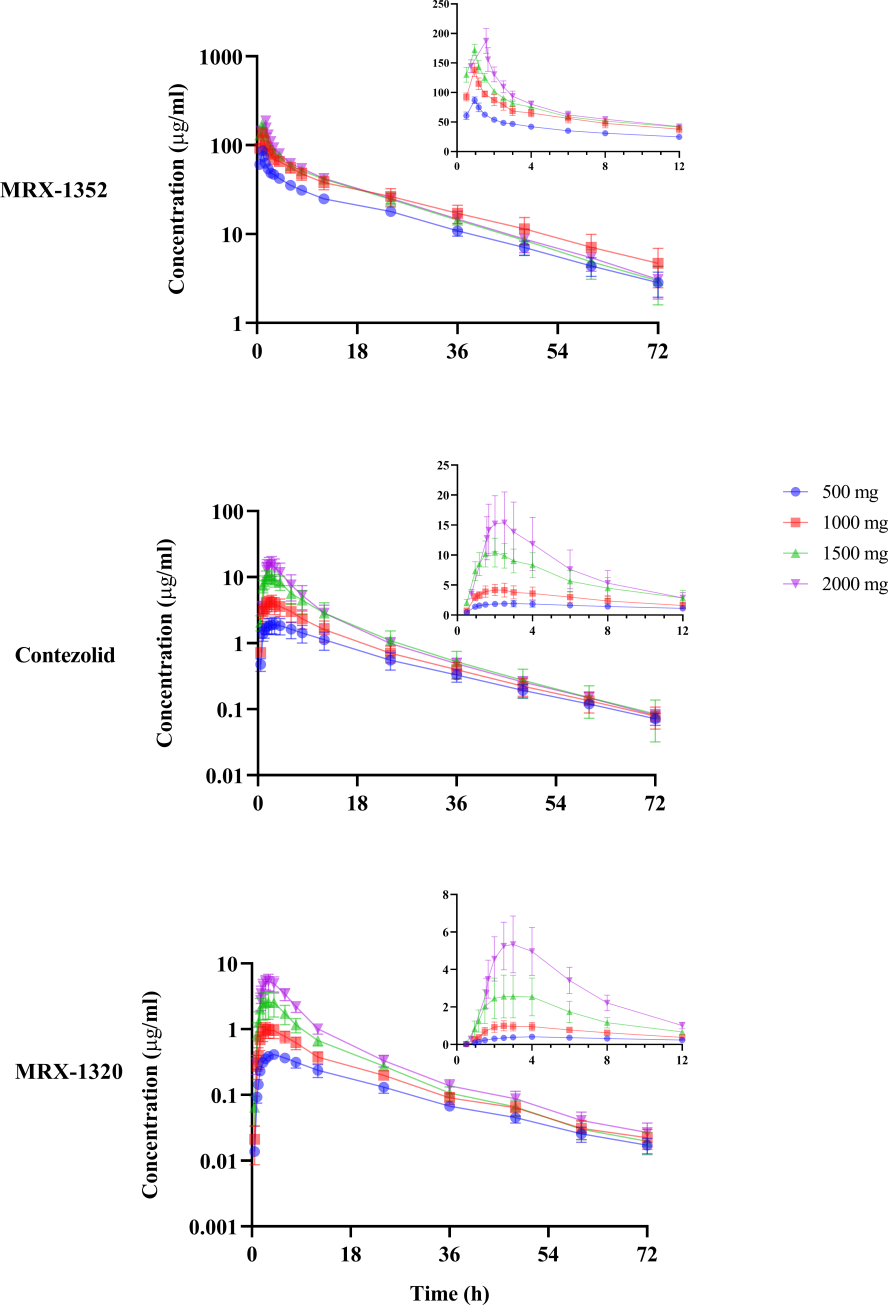
Mean plasma concentration-time curves of MRX-1352, contezolid, and MRX-1320 after intravenous administration of single ascending dose (500, 1,000, 1,500, and 2,000 mg) of contezolid acefosamil in healthy Chinese subjects (semi-logarithmic scale in the left lower panels and zoom-in view of linear scale in the upper right panels).

All of the 36 subjects receiving IV administration of contezolid acefosamil were included in the plasma PK analysis. The mean plasma concentration-time curves of the major metabolites (MRX-1352, contezolid, and MRX-1320) after IV SAD of contezolid acefosamil are shown in Fig. 2. The corresponding PK parameters after IV SAD and multiple doses are shown in Table 3 and Table S1, respectively.

**TABLE 3:** Plasma pharmacokinetic parameters of contezolid in healthy Chinese subjects after intravenous and oral administration of contezolid acefosamil^*c*^ ^,^ ^*d*^

	Intravenous administration					Oral administration		
	Single dose				Multiple dose	Single dose		Multiple dose
PK parameter	500 mg (*n* = 6)	1,000 mg (*n* = 6)	1,500 mg (*n* = 6)	2,000 mg (*n* = 8)	2,000/1,000 mg (*n* = 10)	500 mg (*n* = 3)	1,500 mg (*n* = 5)^*a*^	1,500 mg (*n* = 10)
*C*_max_ (mg/L)	1.95 (0.57)	4.26 (1.04)	10.53 (2.39)	15.61 (4.88)	NA	8.66 (2.60)	37.10 (8.66)	NA
*T*_max_ (h)	2.75 (2.00–4.00)	2.25 (2.00–2.50)	2.00 (1.50–2.50)	2.50 (1.67–2.50)	NA	2.50 (2.00–3.00)	2.98 (2.00–3.98)	NA
AUC_0–inf_ (h·mg/L)	40.25 (10.12)	60.48 (16.26)	110.49 (32.68)	129.41 (38.30)	NA	30.44 (7.33)	162.36 (47.08)	NA
*t*_1/2_ (h)	16.74 (2.57)	15.23 (1.62)	13.33 (1.94)	14.09 (1.40)	NA	3.57 (3.06)	15.83 (6.79)	NA
MRT_0-inf_ (h)	21.99 (2.76)	17.75 (2.25)	13.49 (2.75)	11.90 (1.82)	NA	3.56 (0.04)	4.70 (0.49)	NA
*C*_max ,ss_ (mg/L)	NA	NA	NA	NA	12.62 (2.35)	NA	NA	33.16 (8.98)^*b*^
*C*_min,ss_ (mg/L)	NA	NA	NA	NA	1.20 (0.26)	NA	NA	0.90 (0.62)^*b*^
*T*_max, ss_ (h)	NA	NA	NA	NA	2.00 (1.50–2.50)	NA	NA	2.00 (1.50–3.00)^*b*^
AUC_tau, ss_ (h·mg/L)	NA	NA	NA	NA	70.92 (12.20)	NA	NA	134.05 (60.43)^*b*^
*t*_1/2, ss_ (h)	NA	NA	NA	NA	5.75 (4.39)	NA	NA	12.72 (4.28)^*b*^
*R*_ac_ (AUC)	NA	NA	NA	NA	2.20	NA	NA	1.23 (0.28)^*b*^
*R*_ac_ (C_max_)	NA	NA	NA	NA	2.96	NA	NA	1.10 (0.21)^*b*^

^*a*^ Subject 2A206 was excluded from this PK analysis due to nausea and vomiting.

^*b*^ These steady-state data were the mean values of nine subjects because the data of one subject were invalid due to nausea and vomiting on days 9 and 11.

^*c*^ Data are presented as median (minimum, maximum) for *T*_max_ or mean (SD) unless otherwise specified.

^*d*^ AUC, area under the concentration-time curve; AUC_0–inf_, the area under the concentration-time curve from time 0 to infinity; AUC_tau, ss_, the steady-state AUC over a dosing interval; *C*_max_, peak concentration; *C*_max, ss_, steady-state peak concentration; *C*_min, ss_, steady-state trough concentration; MRT, mean retention time; NA, not applicable; PK, pharmacokinetic; *R*_ac_, accumulation ratio; *t*_1/2_, elimination half-life; *t*_1/2,ss_, elimination half-life at steady state; *T*_max_, time to peak concentration; *T*_max, ss_, time to peak concentration at steady state; *λ*_z_, apparent terminal elimination rate constant.

After intravenous infusion of a single ascending dose (500–2,000 mg) of contezolid acefosamil, the median *T*_max_ for the active drug contezolid was 2.00–2.75 h, and the corresponding mean *t*_1/2_ was 13.33–16.74 h. Contezolid exposure increased with the dose of contezolid acefosamil, evidenced by *C*_max_ increasing from 1.95 ± 0.57 to 15.61 ± 4.88 mg/L, and AUC_0–inf_ from 40.25 ± 10.12 to 129.41 ± 38.30 h·mg/L. The AUC_0–inf_ of contezolid showed dose proportionality with contezolid acefosamil (β = 0.88, 90% CI 0.70–1.05) (Table S2). All of the three metabolites of contezolid acefosamil essentially reached steady state on D6 after multiple intravenous doses. The corresponding *T*_max, ss_, *C*_max, ss_, and AUC_tau, ss_ of contezolid were 2.00 h, 12.33 ± 2.28 mg/L, and 71.96 ± 14.77 h·mg/L, respectively. The accumulation ratio of contezolid after the last dose of contezolid acefosamil on D11 was 2.96 based on *C*_max_ and 2.20 based on AUC_0–12 h_, indicating a certain degree of accumulation of contezolid in the human body.

After IV SAD of contezolid acefosamil, the *C*_max_ of MRX-1352 increased from 86.95 ± 5.62 to 187.44 ± 21.11 mg/L, the corresponding AUC_0–inf_ increased from 1184.02 ± 111.41 to 1876.02 ± 226.28 h·mg/L. The *C*_max_ of MRX-1320 increased from 0.42 ± 0.06 to 5.45 ± 1.43 mg/L, and the corresponding AUC_0–inf_ increased from 8.60 ± 1.25 to 46.30 ± 7.51 h·mg/L. None of the above parameters showed linear proportionality with the dose of contezolid acefosamil (Table S2). After IV administration of multiple doses, the C_max, ss_ values of MRX-1352 and MRX-1320 were 123.32 ± 10.92 and 7.07 ± 0.74 mg/L, respectively. The corresponding AUC_tau, ss_ of MRX-1352 was 372.76 ± 42.94, while the AUC_tau, ss_ of MRX-1320 was not calculable. After IV administration, contezolid acefosamil was only detected in the 2,000-mg dose cohort. Contezolid acefosamil rapidly entered bloodstream after administration, with a *C*_max_ of 1.05 mg/L, AUC_0–*t*_ of 0.72 h·mg/L, and *T*_max_ of 1.50 h, indicating rapid elimination after reaching peak concentration at the end of infusion. Contezolid acefosamil was nearly undetectable at 1.67 h after administration.

**Fig 3:**
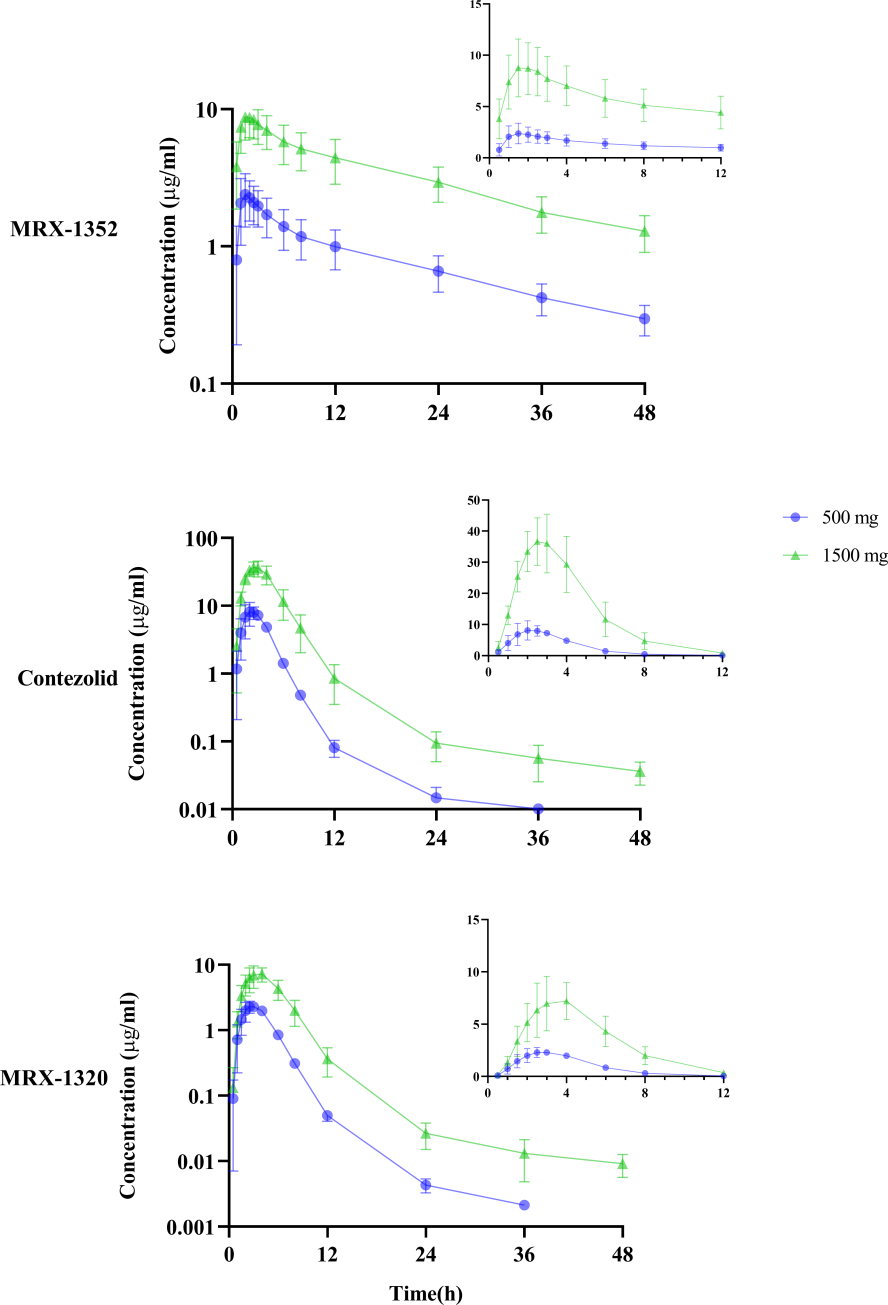
Mean plasma concentration-time curves of MRX-1352, contezolid, and MRX-1320 after oral administration of single ascending dose (500 and 1,500 mg) of contezolid acefosamil in healthy Chinese subjects [semi-logarithmic scale in the left lower panels and zoom-in view of linear scale (0–12 h) in the upper right panels].

One subject in the 1,500-mg cohort of oral SAD study was not included in the PK analysis set due to vomiting. The vomiting might have some effect on the reliability of the concentration data. One subject in the 1,500-mg multiple-dose cohort was also excluded from PK analysis set due to vomiting on D9 and D11. The mean plasma concentration-time curves of MRX-1352, contezolid, and MRX-1320 after oral SAD of contezolid acefosamil are shown in Fig. 3. The PK parameters of contezolid, MRX-1352, and MRX-1320 after oral SAD and multiple doses of contezolid acefosamil are shown in Table 3 and Table S1, respectively.

Both the *C*_max_ and AUC_0–inf_ of contezolid increased with the oral dose of contezolid acefosamil. All of the three metabolites essentially reached steady state on D5 after multiple oral doses of 1,500-mg contezolid acefosamil. The accumulation ratio of contezolid after the last dose on D11 was 1.07 based on *C*_max_ and 1.18 based on AUC_0–12 h_, indicating no accumulation of contezolid in the human body.

### Urinary PK parameters

The urinary excretion and PK parameters of MRX-1352, contezolid, and MRX-1320 in subjects receiving IV and oral SAD and multiple doses of contezolid acefosamil are shown in Table 4.

**TABLE 4:** Urine pharmacokinetic parameters of MRX-1352, contezolid, and MRX-1320 in healthy Chinese subjects after intravenous and oral administration of contezolid acefosamil^*b*^ ^,^ ^*c*^

	Intravenous administration				Oral administration	
	Single dose			Multiple dose	Single dose	Multiple dose
PK parameter	1,000 mg (*n* = 6)	1,500 mg (*n* = 6)	2,000 mg (*n* = 8)	2,000/1,000 mg (*n* = 10)	1,500 mg (*n* = 5)^*a*^	1,500 mg (*n* = 9)^*a*^
MRX-1352						
UA_0–48 h_ (mg)	NA	NA	NA	NA	0.40 (0.17)	0.24 (0.14)
UA_0–72 h_ (mg)	17.63 (4.53)	34.24 (13.08)	65.53 (43.99)	32.86 (13.86)	NA	NA
CL_r_ or CL_r_/*F* (L/h)	0.01 (0.00)	0.02 (0.01)	0.04 (0.03)	0.09 (0.04)	0.00 (0.00)	0.00 (0.00)
Contezolid						
UA_0–48 h_ (mg)	NA	NA	NA	NA	20.68 (4.48)	21.91 (8.59)
UA_0–72 h_ (mg)	167.95 (9.19)	229.55 (25.96)	348.31 (126.19)	164.62 (11.78)	NA	NA
CL_r_ or CL_r_/F (L/h)	3.05 (0.89)	2.24 (0.62)	2.83 (1.03)		0.13 (0.03)	0.16 (0.04)
MRX-1320						
UA_0–48 h_ (mg)	NA	NA	NA	NA	573.22 (115.10)	703.73 (135.18)
UA_0–72 h_ (mg)	288.65 (23.11)	426.31 (58.68)	590.63 (70.16)	365.56 (23.23)	NA	NA
CL_r_ or CL_r_/*F* (L/h)	20.48 (3.60)	15.93 (1.88)	13.26 (2.78)	6.21 (1.08)	15.10 (4.05)	13.58 (2.12)

^*a*^ Subject 2B106 was excluded from this PK analysis due to nausea and vomiting on day 11.

^*b*^ The data are presented as mean (SD) unless otherwise specified. CL_r_/*F* is the PK parameter for oral administration.

^*c*^ *F*, bioavailability; CL_r_, renal clearance; NA, not applicable; UA_0–48 h_, cumulative urinary excretion from the time of dosing to 48 h; UA_0–72 h_, cumulative urinary excretion from the time of dosing to 72 h.

**Fig 4:**
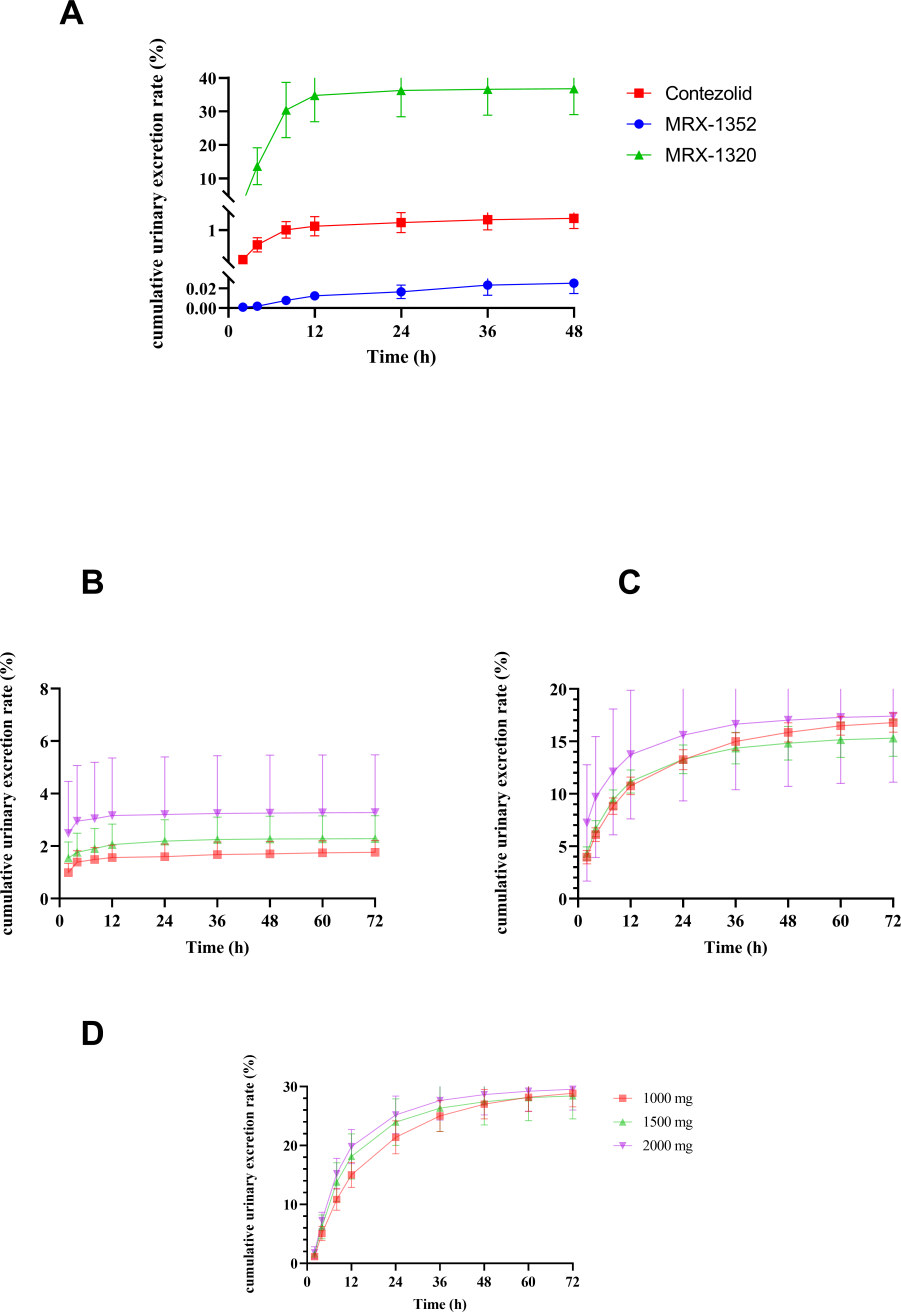
The cumulative urinary excretion rate-time curves of MRX-1352, contezolid, and MRX-1320 after intravenous or oral administration of single ascending dose of contezolid acefosamil. (**A**) For MRX-1352, contezolid, and MRX-1320 after an oral dose of 1,500-mg contezolid acefosamil. (**B**) For MRX-1352 after intravenous administration of single ascending dose (1,000, 1,500, and 2,000 mg) of contezolid acefosamil. (**C**) For contezolid after intravenous administration of single ascending dose (1,000, 1,500, and 2,000 mg) of contezolid acefosamil. (**D**) For MRX-1320 after intravenous administration of single ascending dose (1,000, 1,500, and 2,000 mg) of contezolid acefosamil.

After IV SAD of 1,000- to 2,000-mg contezolid acefosamil in healthy subjects, the UA_0–72 h_ values of MRX-1352, contezolid, and MRX-1320 were 17.63 ± 4.53 to 65.53 ± 43.99 mg, 167.95 ± 9.19 to 348.31 ± 126.19 mg, and 288.65 ± 23.11 to 590.63 ± 70.16 mg, respectively. The corresponding 0- to 72-h cumulative urinary excretion of MRX-1352, contezolid, and MRX-1320 accounted for 1.76%–3.28%, 15.30%–17.42%, and 28.42%–29.53% of the administered contezolid acefosamil dose, respectively (Table 4; Fig. 4). After the last dose of multiple IV administration, the UA_0–72 h_ of each metabolite was consistent with that after single-dose administration.

Urine samples from the subjects were only collected up to 48 h after oral administration of 1,500 mg in healthy subjects. The UA_0–48 h_ values of MRX-1352, contezolid, and MRX-1320 were 0.40 ± 0.17, 20.68 ± 4.48, and 573.22 ± 115.10 mg, respectively. The corresponding cumulative urinary excretion of MRX-1352, contezolid, and MRX-1320 accounted for 0.02%, 1.38%, and 38.21% of the administered contezolid acefosamil dose, respectively (Table 4; Fig. 4). After the last dose of multiple oral administration of contezolid acefosamil, the UA_0–48 h_ of each metabolite was consistent with that after single-dose administration.

### Population PK (popPK) analysis

A total of 990 contezolid plasma concentrations from 36 subjects in the IV dose cohorts were included in this popPK analysis. Based on the popPK study of a similar drug conducted by Li et al. (19) and the *in vivo* metabolic profiles of active drug contezolid described in this study, the final model for contezolid acefosamil adopted a simplified two-compartment model for IV administration as the basic structural model. The exponential model was used to investigate the inter-individual variability, and the mixed residual error model was used to investigate the intra-individual variability. The statistically significant covariate, body weight on clearance (CL), was retained in the final model. The final model parameter estimates and the estimation precision are shown in Table S3. The final model is expressed in formulas 1–4, where the volumes of distribution of the central and peripheral compartments, *V* and *V*2, were calculated with formulas 1 and 2. The CL from central to peripheral compartment (Q) was calculated with formula 4, and the CL was calculated with formula 3 (body weight was included as a covariate).


(1) V=8.45 × Exp(ηV)

(2) V2=5.95 × Exp(ηV2)

(3) $$CL=14.83\;\times \;Exp(\eta CL)\;\times \;(WT\;/\;median(WT{)}^{0.86})$$

(4) Q=1.39 ×  Exp(ηQ)

In the above formulas, *η* represents inter-individual variability.

### PopPK model diagnosis and assessment

As shown in Fig. S1, no remarkable deviations were found in the goodness-of-fit (GOF) plots of observed concentrations vs population predicted concentrations (PRED) and individual predicted concentrations, and conditional weighted residuals vs time or PRED. Bootstrap method was adopted to evaluate the stability of the model. All 500 bootstrap iterations successfully converged, with a success rate of 100%. As shown in Table S3, the medians of parameters calculated by bootstrap were generally consistent with the mean parameters predicted by the model. The parameters predicted by the model all fell within the 95% CIs of the bootstrap-calculated parameters, indicating the good stability of the model. The visual predictive check (VPC) results (Fig. S2) based on 500 simulations of contezolid plasma concentrations demonstrated that the final model had good predictive performance and could well describe the PK characteristics of the active ingredient contezolid in the human body after administration of contezolid acefosamil.

In the final popPK model, *V*, *V*2, *Q*, and CL were 8.45 L, 5.95 L, 1.39 L/h, and 14.83 L/h, respectively. The model included body weight as a covariate, which showed a significant effect on CL. Other covariates including demographic (age, sex, and BMI) or laboratory tests did not show a significant effect on the exposure of contezolid. The covariate analysis revealed that the exposure of the active drug contezolid decreased as body weight increased. A sensitivity analysis was conducted to evaluate the impact of body weight on the exposure of the active substance contezolid. The popPK simulation consisted of 500 virtual subjects, with a median weight of 60 kg (range: 40–120 kg). Participants were assigned to one of the three weight categories: 40–59 kg, 60–79 kg, and 80–120 kg. The dosing regimen for simulation was a loading dose of IV infusion of 2,000-mg contezolid acefosamil followed by a maintenance dose of 1,000 mg, administered twice daily for 14 consecutive days. However, the results of the sensitivity analysis indicated that the exposure (AUC_0–24 h_) of contezolid was similar among different body weight groups, and the AUC_0–24 h_ ratio among body weight groups was within the range of 0.85–0.93 (Fig. S3; Table S4), suggesting that the impact of body weight on the exposure of contezolid was limited. Thus, it is not necessary to adjust the dose of contezolid acefosamil based on the body weight of subjects.

## DISCUSSION

The primary objective of this study was to evaluate the safety, tolerability, and PK profiles of contezolid acefosamil in healthy Chinese adults. Overall, contezolid acefosamil was well-tolerated after IV or oral SAD and multiple doses. All TEAEs were mild and resolved spontaneously without discontinuation or treatment. No deaths, SAEs, severe AEs, or AEs leading to early withdrawal were reported.

After oral administration of contezolid acefosamil, the incidence of study drug-related AEs increased with single-dose escalation and multiple doses (80.0% in multiple-dose cohort and 11.1% in single-dose cohorts). The most common study drug-related AEs in the oral cohorts were gastrointestinal symptoms, including nausea, vomiting, and abdominal distension, which are consistent with the known AEs of oxazolidinone drugs (20). The phase I study results of contezolid reported by Eckburg et al. (21) indicated that the time to peak of contezolid was 2–3 h after oral administration of a single dose of contezolid, and no accumulation of contezolid was noted after multiple oral doses (accumulation ratio: 1.02–1.06). These PK characteristics of contezolid acefosamil tablets after oral administration were similar to those of contezolid. Following a single oral dose of 1,500-mg contezolid acefosamil tablets, the primary form excreted in urine was MRX-1320, with very low levels of contezolid and MRX-1352, whose cumulative urinary excretion within 0–72 h accounted for only 0.02% and 1.38% of the administered dose of contezolid acefosamil, respectively. This finding was similar to that of contezolid tablets (0- to 48-h cumulative urinary excretion of contezolid accounted for 0.8%-2.3% of the administered dose) (21).

The results of this study indicate that the safety, tolerability, and PK characteristics of oral contezolid acefosamil are similar to those of contezolid (21). Oral contezolid acefosamil is a prodrug of contezolid, which has been on the market. In the future sequential treatment from intravenous to oral administration, it will be replaced by contezolid.

Drug-related AEs were less common with IV contezolid acefosamil (11.1%) compared with contezolid acefosamil tablets (47.4%), especially in gastrointestinal symptoms. In addition, drug-related AEs did not tend to increase with IV dose escalation or multiple doses. There is no infusion site pain, swelling, or paraesthesia reported in this study, which were the most common AEs in the phase I trial of tedizolid injection (22, 23). Therefore, this drug has a better local safety profile compared with tedizolid.

The typical adverse reactions which limit the clinical use of oxazolidinone antibiotics include thrombocytopenia, leukopenia, pancytopenia, and anemia. Tedizolid, as a second-generation oxazolidinone antibiotic, may also cause thrombocytopenia after 6-day treatment (24). When comparing the risk of thrombocytopenia between linezolid and tedizolid in real-world comparative safety studies using FDA Adverse Events Reporting System data, no significant differences were observed between these two antibiotics (10). Contezolid, as the latest oxazolidinone antibiotic, demonstrated signiﬁcantly lower incidence of leukopenia (0.3% vs 3.4%) and thrombocytopenia (0% vs 2.3%) than linezolid (25). Therefore, the present study also focused on hematology-related AEs. There was no myelosuppression, especially thrombocytopenia after oral doses in this study. Three subjects experienced leukopenia in the intravenous dose cohort but recovered in a few days without treatment. It is important to note that no thrombocytopenia was reported after IV administration of a single dose or multiple doses of contezolid acefosamil. In a phase II clinical study, hematology-related AEs were reported less frequently in the contezolid acefosamil group compared with the linezolid group after treatment for 10–14 days, specifically neutropenia (0% vs 3.7%) and thrombocytopenia (2.5% vs 5.2%) (unpublished data). The hematology-related safety of contezolid acefosamil injection after treatment for 10 days or a longer time should be monitored continuously in future studies.

Since long-term use of linezolid can cause retinopathy (26 – 28), the subjects in the IV or oral multiple-dose cohorts of this study received extra examinations on visual acuity, color discrimination, visual field, and fundus, and no related AEs were found. But further investigations in global phase III trial and post-marketing studies are required to confirm this safety profile.

In this study, the parent drug contezolid acefosamil was only measured in the IV SAD cohorts because contezolid acefosamil concentration was below the lower limit of quantitation at all sampling time points in all of the oral cohorts according to the results of the phase I study in the US. The plasma concentrations of metabolites increased with the IV dose of contezolid acefosamil. The parent drug contezolid acefosamil was undetectable *in vivo* within the dose range of 500–1500 mg but detected in the 2,000-mg cohort after a single-dose IV administration. After IV administration of 2,000 mg, contezolid acefosamil was rapidly converted to MRX-1352, which was gradually transformed into the active metabolite contezolid, and then degraded into MRX-1320. The *T*_max_ of contezolid acefosamil was 1.50 h, indicating rapid elimination after reaching peak concentration at the end of IV infusion. The parent drug contezolid acefosamil was nearly undetectable 1.67 h after administration. Therefore, after a single-dose IV administration, the plasma concentration of contezolid acefosamil was much lower than that of contezolid acefosamil metabolites. The metabolite with the highest plasma exposure was MRX-1352, followed by contezolid and MRX-1320. After IV administration of multiple doses [loading dose 2,000 mg, followed by 1,000 mg every 12 h (q12h)], a certain degree of accumulation of contezolid was found (accumulation ratio: 2.20–2.96) compared with the 1,000-mg single-dose cohort. The urinary excretion pattern was slightly different for IV contezolid acefosamil, where the primary forms excreted in urine were MRX-1320 and contezolid, and the 0- to 72-h cumulative urinary excretion of MRX-1352 only accounted for 1.76%–3.28% of the administered contezolid acefosamil dose.

At an equivalent dose of 500 mg, the mean ratio of the AUC_0–inf_ of contezolid after oral administration of contezolid acefosamil to the AUC_0–inf_ of contezolid after IV infusion of contezolid acefosamil was 0.76, whereas at a dose of 1,500 mg, this ratio increased to 1.47. The results showed that in the low-dose cohort (500 mg), IV administration of contezolid acefosamil showed a better contezolid conversion rate than oral administration, whereas in the high-dose cohort (1,500 mg), oral administration of contezolid acefosamil showed a higher contezolid conversion rate than IV administration. This phenomenon may be related to the differences in the conversion process of contezolid acefosamil between the different routes of administration, as well as the possible non-linear PK characteristics during the conversion process. The specific underlying mechanism is currently unclear. After multiple IV doses of contezolid acefosamil (loading dose 2,000 mg, followed by 1,000 mg q12h), the steady state contezolid exposure was similar to oral administration of contezolid 800 mg q12h, the clinically effective dose (AUC_tau, ss_: 70.92 ± 12.20 h·ng/mL vs 61.41 ± 7.60 h·ng/mL; C_max, ss_: 12.62 ± 2.35 mg/L vs 14.36 ± 1.91 mg/L) (17). Considering that AUC_0–24 h_/MIC is the best PK/PD index of contezolid, it is predicted that contezolid acefosamil administered intravenously by loading dose 2,000 mg, followed by 1,000 mg q12h can achieve the same efficacy as the multiple oral doses of contezolid 800 mg q12h after meals. This will inform the design of sequential regimen of IV contezolid acefosamil followed by contezolid tablets in the subsequent phase III study.

Considering that contezolid acefosamil injection is a different dosage form important for the patients with severe clinical infections or those who cannot tolerate oral administration, a popPK model of contezolid acefosamil injection in healthy Chinese subjects was constructed. In addition to the data of the IV cohorts in this study, the model also included the relevant data in the second cycle of 2,000-mg single-dose IV cohort (2,000/1,000 mg). The factors affecting the PK of contezolid acefosamil injection were preliminarily examined in the model to support the subsequent development of contezolid acefosamil. This study found that body weight had significant effect on CL, and other covariates had no significant effect on the exposure of contezolid acefosamil injection.

### Conclusion

Both IV and oral administrations of contezolid acefosamil demonstrated good safety and tolerability in healthy Chinese subjects, which can inform the safety, PK, and dose selection for the development of the phase III study of contezolid acefosamil. The new dosage form of contezolid acefosamil may be an effective and safer treatment option for patients with severe multidrug-resistant bacterial infections.

## MATERIALS AND METHODS

### Study design

**Fig 5:**
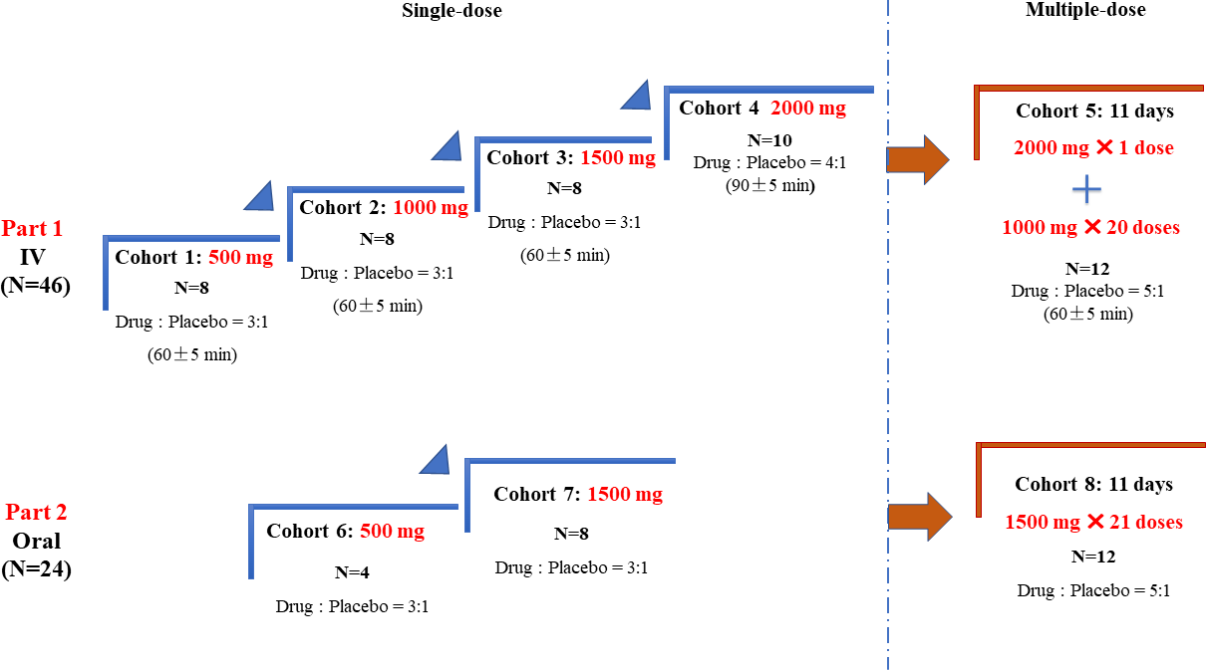
Study design of a phase I clinic trial of contezolid acefosamil in healthy Chinese subjects. IV, intravenous.

This study was a single-center, randomized, double-blind, placebo-controlled, dose escalation phase I clinical trial. A total of 70 subjects were enrolled. As shown in Fig. 5, the study included two parts: IV administration and oral administration. A total of 46 subjects were enrolled in the IV SAD (500, 1,000, 1,500, and 2,000 mg) and multiple dose (loading dose 2,000 mg, followed by 1,000 mg q12h) cohorts. A total of 24 subjects were enrolled in the oral SAD (500 and 1,500 mg) and multiple dose (1,500 mg q12h) cohorts.

The sample size was not based on a formal power calculation. According to the Technical Guidelines for Clinical Pharmacokinetic Research of Chemical Drugs issued by the National Medical Products Administration of China, 8–12 subjects are recommended in each dose cohort for a single- or multiple-dose PK study. An independent statistician performed block randomization, and the random allocation sequences were included in a randomisation envelope according to which the subjects in each dose cohort were allocated. The subjects in each dose cohort were randomised in a ratio of 3:1, 4:1, or 5:1 to receive contezolid acefosamil or placebo.

The first and last doses were administered under fasted conditions (fasted ≥12 h) in all oral cohorts. If ≥1/2 of the subjects in a dose cohort experienced study drug-related grade 2 AEs or ≥1 subject experienced study drug-related grade 3 AEs, the study would be terminated, and the dose would not be further escalated.

### Study participants

The principal investigator fully informed the subjects of the study protocol and possible safety issues. All subjects signed their written informed consent forms before participating in the study. The inclusion criteria include healthy male or female subjects, 18–45 years of age; body weight >50 kg for males and >45 kg for females; BMI 19–26 kg/m^2^; no clinically significant abnormalities in physical examination, vital signs, 12-lead electrocardiogram (ECG), and laboratory tests (including hematology, chemistry, urinalysis, and pregnancy test). The primary exclusion criteria include a history of allergy to any drugs, including oxazolidinone agents; a history of active digestive system diseases within the past 3 months; a history of medication use within the past 2 weeks; a family history of QT interval prolongation syndrome or cardiac conduction abnormalities; and a mean of three QTc intervals >450 ms obtained by 12-lead ECG.

### Administration, sample collection, and determination

Contezolid acefosamil (300 mg/vial, as free acid of contezolid acefosamil, batch no.: 20052051), contezolid acefosamil tablets (250 mg/tablet, as free acid of contezolid acefosamil, batch no.: 1286 A17002), 5% glucose injection (250 mL/bag, placebo, batch no.: S2004098), and contezolid acefosamil tablet placebo (batch no.: R134718001) were provided by Shanghai MicuRx Pharmaceutical Co., Ltd.

In this study, only blood samples were collected for PK analysis in the 500-mg cohorts. Both blood and urine samples were collected for PK analysis in the other dose cohorts. Detailed plasma sampling points for all cohorts are shown in Table S5. According to the sampling points and results of urinary excretion rate in previous phase I clinical studies of contezolid (21, 29), this study was designed to collect urine samples at the following time points: before administration (−12 to 0 h) and at 0–2, 2–4, 4–8, 8–12, 12–24, 24–36, 36–48, 48–60 h (IV cohorts only), and 60–72 h (IV cohorts only) after the start of infusion. Urine samples were collected only before the first dose and after the last dose in multiple-dose cohorts. The blood samples were transferred into blood collection tubes containing sodium heparin and centrifuged within 30 min at 3,000 rpm and 4°C for 10 min. The plasma was aliquoted and stored in a freezer at −70 ± 10°C. The urine samples were aliquoted and stored in a freezer at −70 ± 10°C. High-performance liquid chromatography-tandem mass spectrometry was used to determine the concentrations of contezolid acefosamil and its major metabolites (MRX-1352, contezolid, and MRX-1320) in blood and urine samples. The methodology was established with reference to the contezolid study conducted by Zhao et al. (30). The linear ranges of contezolid acefosamil, MRX-1352, contezolid, and MRX-1320 in plasma were 0.004–2.0, 0.02–20.0, 0.010–10.0, and 0.002–2.0 mg/L, respectively. The linear ranges of MRX-1352, contezolid, and MRX-1320 in urine were 0.02–10.0, 0.02–10.0, and 0.04–20.0 mg/L, respectively. Samples with a concentration below the limit of quantitation were excluded from the analysis. The inter- and intra-run precision and accuracy of the quality control samples met the acceptance criteria for biological samples.

### Safety evaluation

Subjects were confined to the ward and underwent safety evaluation via vital signs monitoring, 12-lead ECG, physical examination, laboratory tests, and AE assessment throughout the entire study. These results were summarized by IV or oral cohorts. The Common Terminology Criteria for Adverse Events (version 5.0) (31) were referred to determine the severity of AEs.

### PK and statistical analyses

Phoenix WinNonlin (version 8.3.1; Certara, USA) was used to calculate the PK parameters of contezolid acefosamil and its major metabolites for each subject with a non-compartmental model. The main PK parameters include peak concentration or steady-state peak concentration (*C*_max_/*C*_max, ss_), time to peak or steady-state time to peak (*T*_max_/*T*_max, ss_), the area under the concentration-time curve from 0 to *t* (AUC_0*–t*_), the AUC from 0 to infinity (AUC_0–inf_), the steady-state dosing interval AUC (AUC_tau, ss_), the clearance or the steady-state clearance (CL/CL_ss_), volume of distribution or steady-state volume of distribution (*V*_*z*_/*V*_ss_), elimination half-life (*t*_1/2_), cumulative urinary excretion (UA_0–24 h_, UA_0–48 h_, and UA_0–72 h_), renal clearance and accumulation ratio. The power model was used to explore whether AUC_0–inf_, AUC_0–_ *_t_*, and C_max_ showed a linear relationship with dose after a single IV or oral dose administration (formula 5):

Formula 5: Ln(*y*) = *β*0 + β1*Ln(dose),

where *y* is the PK parameter including AUC_0–inf_, AUC_0–*t*_, and *C*_max_; *β*0 is the intercept; *β*1 is the slope. When *β* 95% CI contained 1, it was considered that AUC_0–inf_ had a linear relationship with dose. A difference was considered to be statistically significant when *P* < 0.05. Statistical analysis was conducted using SAS version 9.2.

### popPK analysis

In this study, a popPK model of contezolid acefosamil in healthy Chinese subjects was constructed, which preliminarily investigated the factors affecting the PK of contezolid acefosamil. The data of the popPK model for contezolid acefosamil were derived from healthy Chinese subjects (all data of the IV cohorts), and the active ingredient contezolid was mainly considered during the modeling. The model was constructed using the non-linear mixed effects model of Phoenix (Certara, LP, Princeton, NJ, USA). The basic structural model mainly investigated the one-, two-, and three-compartment models. The exponential model was adopted for examining the inter-individual variability. The additive, proportional, and mixed residual error models were used for investigating the intra-individual variability. The covariates to be included were preliminarily screened by correlation analysis on covariates and various system parameters. The covariates include demographic variables (age, body weight, gender, BMI, and body height) and clinical laboratory tests (hemoglobin, platelet count, albumin, total protein, hematocrit, alanine aminotransferase, alkaline phosphatase, aspartate aminotransferase, total bilirubin, direct bilirubin, and creatinine). Forward selection and backward elimination methods were adopted in the covariate screening process. A covariate was selected when the objective function value (OFV) decreased by more than 6.63 after forward selection. A covariate was eliminated from the model when the OFV increased by less than 10.83 after backward elimination. The final model was determined according to parameter estimation precision, GOF plot, and OFV.

### Final model assessment

The superiority/inferiority of the final model was validated using the non-parametric bootstrap method and VPC. The bootstrap method involved repeated random sampling with replacement and modeling of the generated data sets to obtain the PK parameters for each data set. The stability of the model was evaluated by calculating the medians and 95% CIs of the PK parameters and comparing them to the estimated values of the final model. VPC involved the generation of virtual sample data sets through simulation, construction of the 95% prediction intervals for observed and predicted values, and estimation of the 5%, 25%, 50%, 75%, and 95% quantiles of the observed and predicted values. The predictive accuracy of the model was evaluated by comparing the overlap between the observed and predicted values.

## SUPPLEMENTAL MATERIAL

Supplemental file 1 aac.00796-23-s0001.pdf Supplemental Material.

ASM does not own the copyrights to Supplemental Material that may be linked to, or accessed through, an article. The authors have granted ASM a non-exclusive, world-wide license to publish the Supplemental Material files. Please contact the corresponding author directly for reuse.
