# Supplementary material for: A phase I study of the safety, tolerability, and pharmacokinetics of contezolid acefosamil after intravenous and oral administration in healthy Chinese subjects
Source: Antimicrob Agents Chemother. 2023 Oct 30;67(11):e00796-23. doi: 10.1128/aac.00796-23 (PMC10648862; doi:10.1128/aac.00796-23)
Supplement: Supplemental file 1 — Supplemental Material. [file aac.00796-23-s0001.pdf]

Supplemental Material

TABLE S1 Plasma pharmacokinetic parameters of MRX-1352 and MRX-1320 in healthy Chinese subjects after intravenous and oral administration of contezolid acefosamil

	**Intravenous administration**					**Oral administration**		
	**SAD**				**Multiple dose**	**SAD**		**Multiple dose**
**PK parameter**	**500 mg (*n*=6)**	**1000 mg (*n*=6)**	**1500 mg (*n*=6)**	**2000 mg (*n*=8)**	**2000/1000 mg (*n*=10)**	**500 mg (*n*=3)**	**1500 mg (*n*=5)[a]**	**1500 mg (*n*=10)**
**MRX-1352**								
C_{max} (mg/L)	86.95 (5.62)	138.45 (11.23)	172.22 (9.53)	187.44 (21.11)	NA	2.42 (0.95)	8.27 (2.14)	NA
T_{max} (h)	0.98 (0.95, 1.03)	1.03 (1.02, 1.07)	1.01 (0.97, 1.05)	1.50 (1.43, 1.57)	NA	1.50 (1.50, 2.00)	1.50 (1.00, 2.00)	NA
$AUC_{0\text{-}inf}$ (h*mg/L)	1184.02 (111.41)	1845.93 (407.33)	1777.11 (216.16)	1876.02 (226.28)	NA	46.19 (13.46)	178.81 (45.36)	NA
$t_{1/2}$ (h)	18.19 (2.82)	18.49 (3.10)	15.37 (2.06)	15.39 (1.94)	NA	20.47 (1.32)	19.76 (1.58)	NA
λ_z (1/h)	0.04 (0.01)	0.04 (0.01)	0.05 (0.01)	0.05 (0.01)	NA	0.03 (0.00)	0.04 (0.00)	NA
MRT (h)	24.97 (3.68)	25.30 (4.05)	19.92 (3.43)	19.44 (2.89)	NA	28.53 (1.97)	28.28 (1.91)	NA
$C_{max,ss}$ (mg/L)	NA	NA	NA	NA	121.91 (12.52)	NA	NA	10.91 (3.36)[b]
$C_{min,ss}$ (mg/L)	NA	NA	NA	NA	4.05 (1.32)	NA	NA	5.63 (1.49)[b]
$T_{max,ss}$ (h)	NA	NA	NA	NA	0.99 (0.95, 1.07)	NA	NA	1.50 (0.50, 2.50)[b]
$AUC_{tau,ss}$ (h*mg/L)	NA	NA	NA	NA	365.48 (46.62)	NA	NA	96.98 (28.50)[b]
$t_{1/2,ss}$ (h)	NA	NA	NA	NA	4.20 (3.17)	NA	NA	20.65 (2.43)[b]
**MRX-1320**								
C_{max} (mg/L)	0.42 (0.06)	1.03 (0.26)	2.65 (1.10)	5.45 (1.43)	NA	2.40 (0.39)	7.68 (2.53)	NA
T_{max} (h)	4.00 (3.00, 4.00)	2.75 (2.00, 4.00)	3.00 (2.00, 4.00)	2.75 (2.50, 4.00)	NA	3.00 (2.50, 4.00)	3.98 (2.50, 4.00)	NA
$AUC_{0\text{-}inf}$ (h*mg/L)	8.60 (1.25)	14.93 (2.55)	27.45 (5.28)	46.30 (7.51)	NA	10.54 (1.29)	39.58 (9.36)	NA
$t_{1/2}$ (h)	16.35 (3.17)	15.12 (2.11)	12.29 (1.24)	13.94 (1.42)	NA	2.90 (0.49)	10.95 (5.87)	NA
λ_z (1/h)	0.04 (0.01)	0.05 (0.01)	0.06 (0.01)	0.05 (0.00)	NA	0.24 (0.05)	0.09 (0.06)	NA
MRT (h)	22.88 (3.55)	18.64 (2.91)	13.29 (3.08)	11.16 (1.74)	NA	4.36 (0.25)	5.54 (0.62)	NA
$C_{max,ss}$ (mg/L)	NA	NA	NA	NA	8.48 (1.14)	NA	NA	10.21 (1.32)[b]
$C_{min,ss}$ (mg/L)	NA	NA	NA	NA	1.11 (0.28)	NA	NA	0.44 (0.20)[b]

$T_{max,ss}$ (h)	NA	NA	NA	NA	4.00 (3.00, 4.00)	NA	NA	2.50 (2.00, 4.00)[b]
$AUC_{tau,ss}$ (h*mg/L)	NA	NA	NA	NA	55.72 (8.58)	NA	NA	49.09 (10.21)[b]
$t_{1/2,ss}$ (h)	NA	NA	NA	NA	20.28 (15.62)	NA	NA	9.51 (4.32)[b]

Data are presented as median (minimum, maximum) for T_{max} or mean (SD) unless otherwise specified.

[a] Subject 2A206 was excluded from this PK analysis due to nausea and vomiting.

[b] These steady-state data were the mean values of 9 subjects because the data of one subject were invalid due to nausea and vomiting on Day 9 and Day 11.

AUC, area under the concentration-time curve; $AUC_{0\text{-}inf}$, the area under the concentration-time curve from time 0 to infinity; $AUC_{tau,ss}$, the steady-state AUC over a dosing interval; C_{max}, peak concentration; $C_{max,\ ss}$, steady-state peak concentration; $C_{min,\ ss}$, steady-state trough concentration; MRT, mean retention time; NA, not applicable; PK, pharmacokinetic; $t_{1/2}$, elimination half-life; $t_{1/2,ss}$, elimination half-life at steady state; T_{max}, time to peak concentration; $T_{max,\ ss}$, time to peak concentration at steady state; λ_z, apparent terminal elimination rate constant.

TABLE S2 Power model analysis to characterize the effect of dosage on the main pharmacokinetic parameters of MRX-1352, contezolid, MRX-1320 (M2) after intravenous administration of single ascending dose (500-2000 mg) of contezolid acefosamil

**Metabolite**	**Parameter**	**Dose range**	**N**	**Slope (β1)**	**90% *CI***
MRX-1352	Ln(C_{max})	500-2000 mg	26	0.56	(0.50, 0.62)
	Ln(AUC_{0-t})	500-2000 mg	26	0.34	(0.25, 0.43)
	Ln(AUC_{0-inf})	500-2000 mg	26	0.31	(0.21, 0.41)
Contezolid	Ln(C_{max})	500-2000 mg	26	1.54	(1.35, 1.73)
	Ln(AUC_{0-t})	500-2000 mg	26	0.90	(0.72, 1.08)
	Ln(AUC_{0-inf})	500-2000 mg	26	0.88	(0.70, 1.05)
MRX-1320	Ln(C_{max})	500-2000 mg	26	1.84	(1.63, 2.05)
	Ln(AUC_{0-t})	500-2000 mg	26	1.24	(1.11, 1.37)
	Ln(AUC_{0-inf})	500-2000 mg	26	1.21	(1.08, 1.34)

Slope was obtained from the power model: Ln(PK) = Intercept + slope × Ln(X/Xmedian), where PK indicates pharmacokinetic parameters, X means independent variable (dosage), and Xmedian is the median of independent variable. CI, confidence interval.

TABLE S3 Final model parameter estimates and bootstrap results

		Final Model			Bootstrap	
Typical value	Unit	Estimate	CV%	Omega	Estimate (median)	95% *CI*
V	L	8.45	16.27	0.21	10.15	3.16-48.59
V2	L	5.95	5.69	0.08	6.6	5.54-12.59
CL	L/hr	14.83	0.48	0.05	15.2	13.86-17.44
Q	L/hr	1.39	16.63	NA	1.7	0.42-85.85
CLWT		0.86	2.95	NA	0.91	0.15-1.53
PROP		0.17	1.63	NA	0.17	-0.65
add		251.95	5.67	NA	121.65	37.06-4977.79

add, additive residual variation; CL, clearance; CLWT, body weight effect on CL; CV, coefficient of variation; PROP, proportional residual variation; Q, inter-compartment clearance between central compartment and peripheral compartment; NA, not applicable; V, apparent volume of the central compartment; V2, apparent volume of the peripheral compartment.

Table S4 The simulated AUC0-24h of contezolid in terms of body weight on Day 1 and Day 14 after IV infusion of 2000 mg contezolid acefosamil over 1 h followed by a maintenance dose of 1000 mg, administered twice daily for 14 consecutive days based on the population PK model.

Day	PK parameter	Body weight	Mean AUC_{0-24}	CV%	AUC_{0-24} Ratio (High/Low)
Day 1	AUC_{0-24} (mg*h/L)	Low: 40-59 kg	229.17	31.76	0.93
		Medium: 60-79 kg	215.68	34.15	
		High: 80-120 kg	213.25	50.48	
Day 14	AUC_{0-24} (mg*h/L)	Low: 40-59 kg	195.85	37.57	0.85
		Medium: 60-79 kg	180.87	56.90	
		High 80-120 kg	165.49	57.79	

AUC_{0-24h}, area under the concentration-time curve from 0 to 24h; CV, coefficient of variation.

TABLE S5 Timetable for blood sample collection

Administration	Number of doses	Cohort	Schedule for blood collection
Intravenous	SAD	Cohort 1: 500 mg<br>Cohort 2: 1000 mg<br>Cohort 3: 1500 mg	Pre-dose (within 60min), 30 min, 60 min (immediately after the completion of IV infusion), 70 min, 1.5, 2, 2.5, 3, 4, 6, 8, 12, 24, 36, 48, 60, and 72 h after the start of IV infusion
		Cohort 4: 2000mg	Pre-dose (within 60 min), 45 min, 90 min (immediately after the completion of IV infusion), 100 min, 2, 2.5, 3, 4, 6, 8, 12, 24, 36, 48, 60, and 72 h after the start of IV infusion
	Multiple dose	Cohort 5:<br>2000/1000 mg	• D1: pre-first dose (within 60min), 30 min, 60 min (immediately after the completion of IV infusion), 70 min, 1.5, 2, 2.5, 3, 4, 6, 8, and 12 h after the start of first IV infusion; 60 min after the start of second IV infusion (immediately after the completion of IV infusion)<br>• D3, D5, D7, D9 (trough concentration): pre-first dose (within 30 min)<br>• D6: pre-first dose (within 30 min), 60 min (immediately after the completion of IV infusion), 1.5, 2, 2.5, 3, 6, and12 h after the start of first IV infusion<br>• D11: the same as for Cohort 1 to Cohort 3
Oral	SAD	Cohort 6: 500 mg<br>Cohort 7: 1500 mg	Pre-dose (within 60 min), 30 min, 1, 1.5, 2, 2.5, 3, 4, 6, 8, 12, 24, 36, and 48 h after administration
	Multiple dose	Cohort 8: 1500 mg	• D1: pre-first dose (within 60 min), 30 min, 1, 1.5, 2, 2.5, 3, 4, 6, 8, 12, 14, 15, and 16 h after the start of the first administration<br>• D3, D5, D7, D9: pre-first dose (within 30 min) (trough concentration); pre-second dose (within 30 min) and 2 h after the second administration<br>• D6: pre-first dose (within 30 min), 1, 1.5, 2, 2.5, 3, 4, 6, and 12 h after the start of the first administration<br>• D11: the same as for Cohort 6 and Cohort 7

IV, intravenous; SAD, single ascending dose.

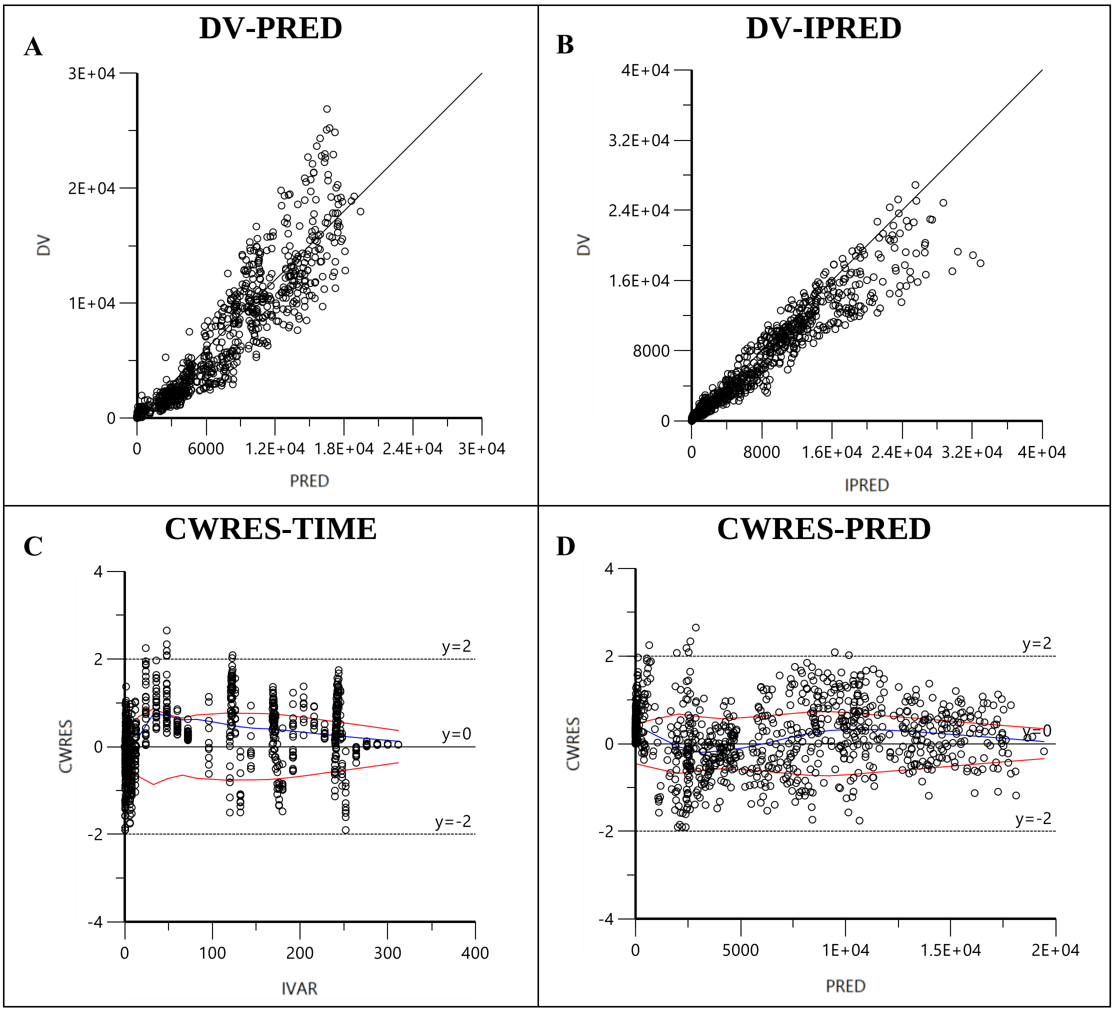

FIG S1 Goodness-of-fit plots for the final population pharmacokinetic model of intravenous contezolid acefosamil. (A) Observed concentration (DV) versus population predicted concentration (PRED). (B) DV versus individual predicted concentration (IPRED). (C) Conditional weighted residuals (CWRES) versus time. (D) CWRES versus PRED. The solid lines in (A) and (B) represent the line of identity, whereas the solid lines in (C) and (D) denote the position where CWRES is equal to 0. The blue lines are regression lines and the red lines represent the distribution of data in (C) and (D).

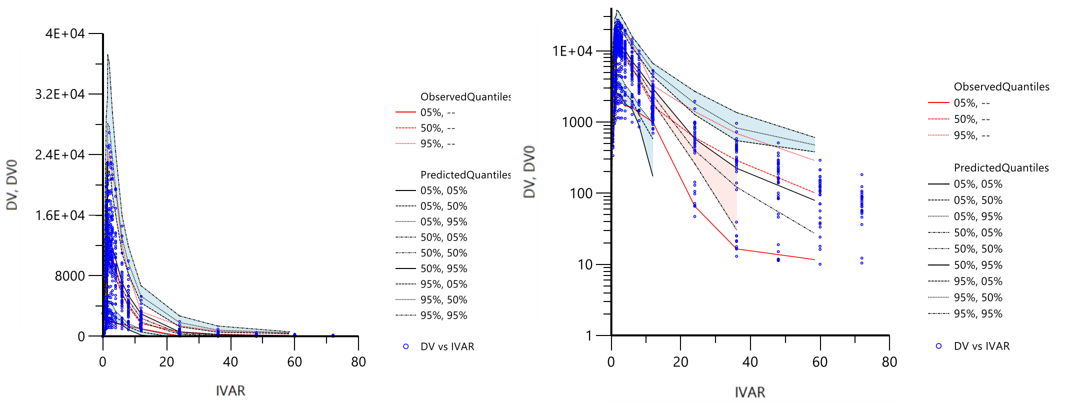

FIG S2 Visual predictive check (VPC) for final population PK model of intravenous contezolid acefosamil in healthy Chinese subjects (linear and semi-log scales). The red lines from top to bottom indicate the 95th, 50th, and 5th percentiles of observed values, respectively. The black lines from top to bottom denote the 95th, 50th, and 5th percentiles of predicted values, respectively. The red and blue shaded areas are the corresponding 90% confidence intervals of the predicted percentiles. DV, observed value (plasma concentration, ng/mL); IVAR, independent variable (time, hr).

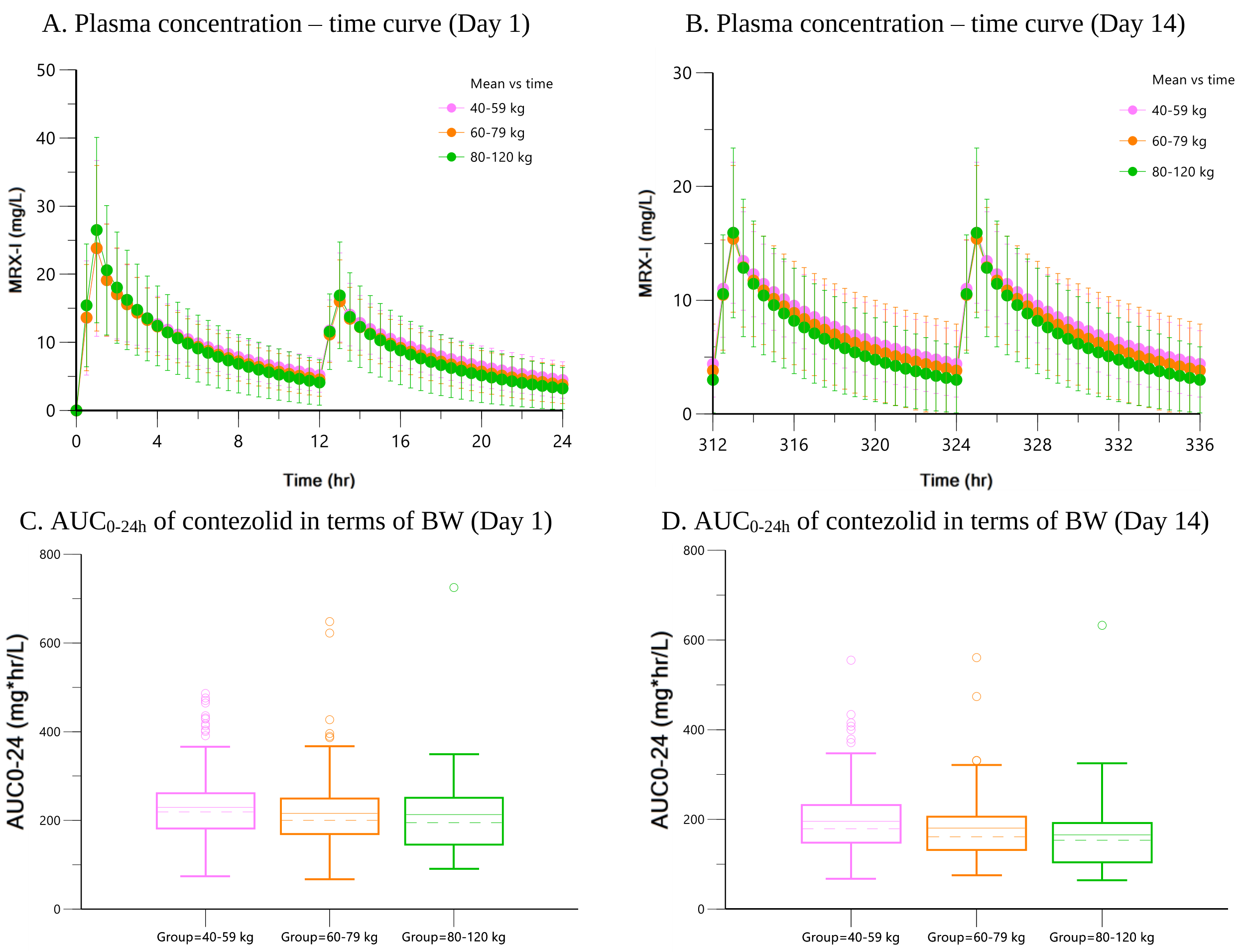

FIG S3 The simulated PK curve and AUC0-24h of contezolid on Day 1 and Day 14 after IV infusion of 2000 mg contezolid acefosamil over 1 h followed by a maintenance dose of 1000 mg, administered twice daily for 14 consecutive days based on the population PK model.
